# Midazolam for Anesthetic Premedication in Children: Considerations and Alternatives

**DOI:** 10.7759/cureus.50309

**Published:** 2023-12-11

**Authors:** Michael Lethin, Matthew R Paluska, Timothy R Petersen, Ricardo Falcon, Codruta Soneru

**Affiliations:** 1 Department of Internal Medicine, Western University of Health Sciences College of Osteopathic Medicine of the Pacific-Northwest, Lebanon, USA; 2 Department of Anesthesiology, Rocky Vista University College of Osteopathic Medicine, Englewood, USA; 3 Office of Graduate Medical Education/Department of Anesthesiology and Critical Care/Department of Obstetrics and Gynecology, University of New Mexico School of Medicine, Albuquerque, USA; 4 Department of Anesthesiology and Critical Care, University of New Mexico School of Medicine, Albuquerque, USA

**Keywords:** pharmacokinetics in children, anxiolytic effect, pediatric surgery, anesthetic agents, preoperative care, pediatric anesthesia, sedation, anxiolysis, pediatric premedication, midazolam

## Abstract

Premedication in anesthesia has long been used to reduce patient anxiety, increase patient compliance, and supplement the overall anesthetic. In pediatric populations, premedication also has the indirect benefits of reducing parental anxiety as well as both the incidence and severity of emergence delirium. Oral midazolam, selected for its ease of administration, short duration of action, and reliable anxiolytic and amnestic effects, has been a favorite choice in this role for decades. The side effect profile of midazolam is also relatively benign, heavily dose-dependent, and easily managed in the perioperative setting. While midazolam appears to be an ideal adjunct in the anesthetic care of pediatric patients, there is a growing body of evidence suggesting prolonged benzodiazepine exposure causes neurodevelopmental changes in infants. This evidence, along with the 2017 Food and Drug Administration (FDA) warning labels for the use of select anesthetic medications, including midazolam in children under the age of three, has led to some debate in the anesthetic community over the continued use of this anesthetic for premedication in pediatric populations. This article aims to educate the reader on the history of midazolam as a premedication agent in pediatric populations and examine the evidence supporting and against its continued use in this role.

## Introduction and background

Preoperative anxiety in children is associated with several adverse postoperative outcomes, including increased pain, sleep disturbances, and negative behavioral changes [1]. There are roughly 4 million pediatric surgeries performed yearly in the United States [2], and more than half of these patients exhibit some form of preprocedural anxiety [3]. Given the prevalence of preoperative anxiety and its association with adverse outcomes, its prevention should be on the mind of every pediatric anesthesiologist when forming a plan of care. Traditionally, options for the treatment of preoperative anxiety have been categorized as either pharmacological or non-pharmacologic. Non-pharmacologic treatments include parental presence at induction of anesthesia (PPIA), distraction techniques such as clowns and electronic devices, and pre-procedural educational programs [4]. Pharmacologic options include benzodiazepines, alpha-2 agonists, ketamine, and melatonin [4]. While oral midazolam has historically been one of the more popular choices in this role because of its ease of administration, short duration of action, favorable side effect profile, and low cost [4], its use has been called into question by critics who point to weak evidence of benefit and the availability of non-pharmacologic alternatives [5,6].

These critiques have been bolstered by studies of the effects of exposure to general anesthesia on lifelong health and behavioral outcomes [7] in conjunction with studies on midazolam’s use for long-term pediatric intensive care unit sedation [8], which have raised questions about its long-term effect on the developing brain. While animal models show a strong relationship between exposure to general anesthetics, including midazolam, and poor neurodevelopmental outcomes [9], this relationship in humans has been more challenging to elucidate. With these two associations in mind, the Food and Drug Administration (FDA) issued new warning labels in 2016 for a variety of anesthetic drugs when used in children less than three years of age [10]. These new labeling requirements include midazolam and warn parents and healthcare providers of its potential neurotoxic effects.

While this new warning highlights the correlation between early childhood exposure to midazolam and negative cognitive and behavioral outcomes, the evidence for a causal relationship remains weak. Only one human study has shown a relationship between midazolam exposure and neurodevelopmental changes in neonates [8]. The relationship between general anesthesia exposure and developmental outcomes is likely multifactorial, as untreated pain, anxiety, and traumatic events in early childhood are all also linked to negative neurobehavioral outcomes later in life [11]. Thus, the question becomes, is the juice worth the squeeze? Do the benefits of the anxiolysis provided by midazolam outweigh the side effects and potential for neurotoxicity, and are there alternatives worth considering? In this article, we examine the evidence for and against using midazolam as a premedication in pediatric surgery.

## Review

Midazolam

Midazolam is a benzodiazepine that binds to postsynaptic γ-aminobutyric acid (GABA)-A receptors within the central nervous system, which in turn increases the flow of chloride ions through the receptor into the postsynaptic neuron [12]. Increased negative charge within the target neuron decreases the likelihood of nerve impulse propagation, leading to a generalized reduction in neuronal activity throughout the central nervous system. This reduction in activity produces the anesthetic, amnestic, and respiratory depression effects benzodiazepines are known for. Midazolam is available for intravenous (IV), intramuscular, intranasal, and oral forms. Following administration, midazolam is distributed hematogenously while bound to albumin, undergoes first-pass metabolism into the less active hydroxylated forms by the cytochrome P450 (CYP) 34A and CYP2B6 enzymes, and is subsequently conjugated for renal excretion [12].

In the setting of procedural pediatric anesthesia, oral dosing is the preferred method of administration due to its ease of administration without the need for IV access and high rate of tolerance in pediatric populations [13]. Oral dosages range from 0.25-1 mg·kg^-1^, with mildly dose-dependent durations of onset and action at 10-20 minutes and 60-90 minutes, respectively. In children over the age of one, half-life ranges from 2.2-6.8 hours, with a clearance range of 3.2-13.3 mL·kg^-1^·min^-1^. If an IV catheter is in place, IV administration offers a shorter onset and duration of action at 2-3 minutes and 45-60 minutes, respectively, with a shorter average half-life of 2.9-4.5 hours [14]. There is a positive correlation between the dose of midazolam and the onset of anxiolysis and it was found that at higher doses (1.0 mg·kg^-1^), a significant number of children achieved satisfactory anxiolysis within 10 minutes [15]. There is a proportion of children who do not respond adequately to midazolam and who may exhibit extreme anxiety. These children are typically significantly younger than those who did respond to midazolam: a higher percentage of nonresponders was observed in children under four years old (24.7%) compared to older children (8.1%) [16].

Midazolam’s primary action on GABA-A receptors, slowing neurotransmission throughout the central nervous system, leads to the desired anxiolytic, sedative, anti-epileptic, and anterograde amnestic effects. The primary undesired side effect is oxygen desaturation, especially when combined with opioids and other depressant medication, which is caused by reduced respiratory drive, relaxation of airway musculature, or a combination of the two. This has been reported in less than 1% of cases and is thought to be dose-dependent, with obstructive sleep apnea as a significant risk factor [17]. Other reactions, including agitation, delirium, and uncontrollable crying, have also been observed. However, the mechanism and prevalence of these paradoxical reactions are less understood, with a widely varying estimated incidence of 1-15% [6].

Overview of perioperative and postoperative behaviors and outcomes

Surgery is a traumatic life event for many children. The strange environment, sights, sounds, and stimuli induce a great deal of fear and anxiety in both the patient and the parent or guardian. This fear and anxiety have been shown to lead to immediate consequences such as increased difficulty with mask induction and even cancellation of surgical procedures in some cases [18]. Increased preoperative anxiety levels have also been linked to delayed adverse outcomes, including increased rates of emergence delirium, as well as increased negative behavioral changes at home in the weeks following the procedure [19]. Exposure to surgical procedures and anesthesia early in life has also been more broadly associated with negative outcomes, including deficits in motor skills, social linguistics, executive function, and behavior.

Evidence for efficacy of midazolam

The effectiveness of midazolam as a preoperative anxiolytic for pediatric patients has been well documented, and more than 80% of anesthesiologists in the US preferred midazolam as a premedication [16]. Many high-quality randomized controlled trials have shown that oral midazolam reduces levels of separation anxiety in both patients and their parents, decreases the incidence of negative preoperative behaviors, including crying, and increases mask acceptance at induction [20]. Evidence also points to the effectiveness of midazolam as an anesthetic adjunct, reducing total propofol and anesthetic gas requirements during induction and maintenance of anesthesia. The effectiveness of oral midazolam in reducing the incidence and severity of emergence delirium needs to be clarified, with some studies showing a reduction in both incidence and severity, some showing a reduction in severity only, and some showing no benefit [21]. The effect on other negative postoperative outcomes is also unclear, with some studies suggesting a reduction in postoperative behavioral disturbance, some showing increased incidence, and some showing no effect at all [22-24]. Some have attributed these conflicting behavioral outcomes to the propensity for midazolam to impair explicit memory formation while preserving implicit memory [5].

No medication is without side effects, however, and midazolam is no exception. The most recent criticism of midazolam follows evidence suggesting that early exposure to the medication can lead to neurodevelopmental changes in neonates [8]. An emerging body of evidence has linked exposure to general anesthesia to lifelong adverse outcomes, including behavioral changes, deficits in fine motor skills, and executive function. This emerging evidence led to the 2017 FDA Drug Safety Communication urging caution in the use of drugs commonly employed in the administration of general anesthesia in pediatric patients below age three [10]. Though no evidence supporting a link between midazolam and these adverse outcomes was included in the report, it was still included in label change guidelines, likely due to its common usage as an anesthetic premedication and sedative in the intensive care unit settings.

Including midazolam in this label has led to the publication of various studies examining the link between neonatal midazolam exposure and neurodevelopmental changes. Animal models have shown a more direct link between total midazolam exposure during the neonatal period and negative behavioral changes but notably have focused on long-term sedation. In addition, the doses used are more than ten or even 100 times the weight equivalent dosing common in the neonatal intensive care unit setting [9,25,26]. One study found a dose-dependent relationship between midazolam exposure and reduction in human hippocampal volume and lower cognitive scores, exacerbated by exposure to surgery [8]. However, it did not report the actual doses administered. The authors of that paper acknowledge many confounding variables preventing them from espousing a definite causal link between midazolam and neurodevelopmental outcomes and urge further investigation.

Alternatives

Alpha-2 Agonists

Oral clonidine and intranasal or buccal dexmedetomidine (2 µg·kg^-1^ (range: 1-4 µg·kg^-1^; maximum 200 µg)) have been considered replacements for midazolam in recent years [27]. These medications share the anxiolytic and sedative properties of midazolam without the amnestic effects and offer a degree of pain relief not provided by benzodiazepines [28]. While they have been shown in meta-analyses to provide levels of sedation and anxiolysis comparable to midazolam, they offer additional benefits in the form of a more robustly documented reduction in emergence delirium [29]. Considerations against their use include significantly increased length of onset and total duration of sedation, with dexmedetomidine taking 10-20 minutes longer to reach peak effect and producing more extended post-anesthesia care unit stays compared to midazolam [27,29]. They have also been shown to increase the incidence of intraoperative bradycardia and hypotension requiring intervention.

N-Methyl-D-Aspartate Antagonists

Ketamine has also been evaluated as a potential alternative to midazolam. It has a faster onset (10-15 minutes) in the oral form (typical oral dosage: 5-8 mg·kg^-1^ or 3 mg·kg^-1^ in combination with midazolam), with sedative, anxiolytic, and analgesic effects [27]. However, its side effect profile includes salivation, increased nausea and vomiting, hallucinations, and increased incidence of emergence delirium, making it less attractive for inclusion at induction of anesthesia.

Opioids

The use of opioids, like oral transmucosal fentanyl citrate (15-20 µg·kg^-1^) [30,31] or oral butorphanol (0.2 mg·kg^-1^ [32]), is practiced in some parts of the world. Oral transmucosal fentanyl is appealing to children for its association with candy being a ‘lollipop’ with a sweetened solid matrix encouraging sucking for transbuccal absorption. These fentanyl ‘lollipops’, according to Howell et al. [30], showed similar anxiolysis to oral midazolam and without postoperative behavioral changes. With the use of fentanyl, there is a concern for pre-operative vomiting. There was also a non-significant difference in postoperative nausea and vomiting to midazolam in the same study.

Some concerns with oral transmucosal fentanyl citrate are that it causes a dose-dependent reduction in respiratory rate and the risk of respiratory depression is present, and in some cases, the postoperative administration of naloxone might be necessary. The typical dose for oral transmucosal fentanyl in pediatric patients is around 5-15 µg·kg^-1^. However, this can vary based on individual patient factors and the specifics of the procedure. Notably, children receiving the higher spectrum of the dosing range (20-25 µg·kg^-1^) displayed a significant decrease in oxygen saturation (SpO_2_) after 30 minutes compared to those on lower doses [31]. This risk underlines the importance of continuous monitoring of oxygen saturation both during the administration of oral transmucosal fentanyl citrate and potentially afterward in the post-anesthesia care unit or on the floor. Interestingly, the occurrence of pruritus was reported to be non-troublesome to either the children or their parents [31].

Another opioid option for anesthetic premedication is butorphanol, a mixed agonist-antagonist at the μ-opioid receptor with agonistic activity at the κ-opioid receptors. One study found that it has more sedation and comparable anxiolysis to oral midazolam with no increase in nausea and vomiting [32]. Fentanyl and other opioids are often favored for rapid onset, short duration of action, and potent analgesic effects. However, the use of opioids in children must be approached with caution due to their potential for respiratory depression (especially in younger children or those with underlying respiratory issues), nausea, and vomiting.

Parental Presence at Induction of Anesthesia

PPIA, as a part of a family-centered approach, is one of the more heavily studied forms of non-pharmacologic anxiolysis prior to pediatric surgery [27]. Proponents argue that by including the patient and their family in the preoperative visit and providing age-appropriate educational materials about the induction and surgical process along with PPIA, overall anxiety levels would decrease, and outcomes would improve. This approach may also reduce the need for premedication, sparing the patient from side effects and reducing the need for preoperative observation. Potential downsides include the additional staff required to supervise parents or guardians during their time in the operating room (OR) and the possibility of parents or guardians interfering with the OR routine or sterility. A 2015 meta-analysis of studies evaluating PPIA found that there is little to no evidence indicating that the family-centered approach and PPIA reduce levels of parental or patient anxiety [33].

Behavioral interventions

Passive video viewing, active video gaming devices, and other distraction-based interventions have also been studied. Providing a familiar alternative point of focus to the child allows them to ignore many of the unknown and anxiety-inducing sights and sounds present during the induction process. Passive video viewing and clowns (doctors or nurses in costume) have been shown to significantly reduce anxiety levels when compared to controls. However, only interactive video gaming is superior to pharmacologic premedication [34]. These video game-based interventions provide a promising alternative to pharmacologic interventions, with no side effects and few drawbacks aside from the cost of the devices. The evidence supporting these interventions is intriguing but limited, with a small number of trials comparing distraction-based intervention to pharmacologic intervention [33]. Table 1 shows a comparison of pharmacologic and non-pharmacologic pediatric anesthesia premedication without IV access.

**Table 1:** Comparison of pharmacologic and non-pharmacologic pediatric anesthesia premedication without IV access GABA: γ-aminobutyric acid; O_2_: Oxygen; OR: Operating room; N/A: Not applicable; NMDA: N-methyl-D-aspartate; PONV: Post operative nausea and vomiting; IV: Intravenous References [17, 27-34]

Medication (Route)	Mechanism of Action	Dose	Benefits	Concerns
Midazolam (Oral)	GABA-A receptor agonist	0.25 - 1 mg·kg^-1^	Anxiolysis, sedation, amnesia, reduced propofol and gas requirements	Potential neurodevelopmental risks, respiratory depression with O_2 _desaturation, especially when combined with opioids and other depressant medication, paradoxical reactions (agitation, delirium, uncontrollable crying)
Clonidine (Oral)	Central α_2_-adrenoceptor agonist	4 mg·kg^-1^ (maximum 200 mg)	Shares the anxiolytic and sedative properties of midazolam without the amnestic effects, offers a degree of pain relief not provided by benzodiazepines	Significantly increased length of onset and total duration of sedation, producing more extended post-anesthesia care unit stays, increased incidence of intraoperative bradycardia and hypotension
Dexmedetomidine (Intranasal or Buccal)	Selective α_2_-adrenoceptor agonist	2 mg·kg^-1^ (range: 1 - 4 mg·kg^-1^; maximum 200 mg)	Intranasal option, shares the anxiolytic and sedative properties of midazolam without the amnestic effects, offers a degree of pain relief not provided by benzodiazepines	Significantly increased length of onset and total duration of sedation, producing more extended post-anesthesia care unit stays, increased incidence of intraoperative bradycardia and hypotension
Ketamine (Oral)	NMDA receptor antagonist primarily	5 - 8 mg·kg^-1^ or 3 mg·kg^-1^ in combination with midazolam	Faster onset (10-15 minutes) in the oral form, sedation, anxiolysis, analgesic effects	Increased incidence of emergence delirium, salivation, increased PONV at high doses, hallucinations
Fentanyl (Transmucosal)	μ-receptor agonist	15-20 µg·kg^-1^	Rapid onset, short duration of action, potent analgesia	Increased PONV, respiratory depression and apnea, opioid dependence
Butorphanol (Oral)	Mixed agonist antagonist at the μ-opioid receptor and agonistic activity at the κ-opioid receptor	0.2 mg·kg^-1^	More sedation and comparable anxiolysis at the time of separation of children from their parents to oral midazolam, potent analgesia, no increase in PONV	Respiratory depression and apnea, opioid dependence
Parental Presence at Induction of Anesthesia	N/A	N/A	Familiar and comforting figure present can alleviate a child’s fears and distress, cost effective for those with limited insurance coverage, non-pharmacological approach avoiding potential side effects	Parenteral distress, maintaining a sterile environment in the OR, logistical challenges accommodating parents in the OR
Behavioral Interventions			Cost-effective for those with limited insurance coverage, video-gaming superior to pharmacologic interventions, non-pharmacological approach avoiding potential side effects, positive and memorable experience for children undergoing surgery	Need for video game console or tablet in the OR, individual specific, maintaining a sterile environment in the OR

## Conclusions

Midazolam is used as a premedication agent in pediatric anesthesia for its proven efficacy in reducing anxiety, facilitating smoother anesthesia induction, and improving the overall experience for pediatric patients undergoing surgery. Binding to GABA-A receptors and reducing neuronal activity provides effective anxiolysis, sedation, and amnesia. However, as FDA warnings and studies highlight, its use is not without concerns, as there is an evolving understanding of the potential neurodevelopmental risks associated with benzodiazepines, which necessitates a reevaluation of its use. As the anesthetic community continues to deliberate on administrating midazolam in light of new evidence, the focus should remain on patient safety and optimizing anesthetic care for the pediatric population.

The challenge in pediatric anesthesia remains to balance the benefits of effective anxiolysis against the potential risks of drug exposure, especially in very young children. While opioids like fentanyl can be used in pediatric anesthesia for their potent analgesic properties, their potential side effects, particularly respiratory depression, make them less ideal as a sole agent for premedication. Alternatives like alpha-2 agonists and NMDA antagonists offer a balance of sedation and analgesia with a more favorable side effect profile. Non-pharmacologic methods should also be considered to minimize medication-related risks. The evolving research will undoubtedly influence future guidelines and practices as clinicians embrace a patient-centered approach that weighs the benefits of midazolam against its risks, emphasizing individualized patient care. As the body of research and evidence grows, so does our understanding of the complex interplay between anesthetics, developing neurology, and long-term outcomes, guiding us toward safer and more effective management of pediatric anesthesia.
